# Genome-wide chromatin accessibility and gene expression profiling during flatfish metamorphosis

**DOI:** 10.1038/s41597-023-02111-4

**Published:** 2023-04-08

**Authors:** Laura Guerrero-Peña, Paula Suarez-Bregua, Alejandro Gil-Gálvez, Silvia Naranjo, Luis Méndez-Martínez, Ricardo Tur, Pablo García-Fernández, Juan J Tena, Josep Rotllant

**Affiliations:** 1 Aquatic Biotechnology Lab, Institute of Marine Research, Spanish National Research Council (IIM-CSIC), Vigo, Spain; 2 Centro Andaluz de Biología del Desarrollo, Consejo Superior de Investigaciones Científicas-Universidad Pablo de Olavide-Junta de Andalucía, Seville, Spain; 3 Nueva Pescanova Biomarine Center, S.L, O Grove, Spain

**Keywords:** Chromatin, Transcriptomics

## Abstract

Metamorphosis is a widely studied post-embryonic process in which many tissues undergo dramatic modifications to adapt to the new adult lifestyle. Flatfishes represent a good example of metamorphosis in teleost fishes. During metamorphosis of flatfish, organ regression and neoformation occur, with one of the most notable changes being the migration of one of the eyes to the other side of the body. In order to create a useful and reliable tool to advance the molecular study of metamorphosis in flatfish, we generated a chromatin accessible atlas as well as gene expression profile during four developmental stages ranging from a phylotypic to a post-metamorphic stage. We identified 29,019 differentially accessible chromatin regions and 3,253 differentially expressed genes. We found stage-specific regulatory regions and gene expression profiles, supporting the quality of the results. Our work provides strongly reproducible data for further studies to elucidate the regulatory elements that ensure successful metamorphosis in flatfish species.

## Background & Summary

Metamorphosis is a widely studied post-embryonic process that allows a larva to adapt to the new demands of adult life. An organism that has to cope with two completely different lifestyles must undergo many morphological, physiological and behavioral changes in a very short period of time^1^. Flatfish undergo one of the most dramatic metamorphoses known in teleosts, in which a symmetrical pelagic larva becomes an asymmetric benthic juvenile^2^, facing internal and external changes such as remodeling of the craniofacial complex^3^, eye migration^4,5^ or redistribution of the skin pigmentation^6^. Although flatfish metamorphosis is well described and the central role of thyroid hormone is well established^7–9^, few data are available on the regulation of possible specific target genes in the context of the gene network responsible for metamorphic remodeling of larval tissues. We emphasize the importance of a thorough understanding of the gene regulation of metamorphosis process in flatfish as they are a group of fish of great economic value in the aquaculture industry.

Metamorphosis, as a biological process based on a perfect orchestration of gene repression and activation for downstream events, requires a high transcriptional regulation^10^. DNA packing is known to leave closed or open chromatin domains that act as biologically inactive or active regions, respectively. Thus, open region of chromatin (i.e., promoters, enhancers and other regulatory elements) are accessible to transcriptional and epigenetic machinery that contributes to the cellular phenotypes^11,12^. Some studies in anurans suggest that transcriptional regulation and chromatin accesibility are key drivers of metamorphosis^13^, and for this reason experimental assays based on transcriptomics and epigenomics are essential to fully understand this process.

ATAC-seq (Assay for Transposase-Accessible Chromatin using sequencing)^11^ is a rapid and sensitive method for probing DNA accessibility that can be used as a proxy for the identification of active genomic events, mainly promoters and enhancers. These data, combined with a transcriptome, generate a perfect set of reliable tools for the study of gene regulation at different strata. Thus, we created, for the first time, an atlas of chromatin accessibility at four developmental stages in a commercially important flatfish, turbot (*Scophthalmus maximus*). We selected a specific time window in turbot development, including in our timeline the phylotypic (3dpf; days post fertilization), pre-metamorphic (15dph; days post hatching), climax of metamorphosis (23dph) and post-metamorphic (37dph) stages. Through a comprehensive analysis of our data, we obtained strong stage-specific concordance results for RNA-seq and ATAC-seq profiling. This complete and reliable dataset will become a useful tool for future studies focused on metamorphosis in different model organisms.

## Methods

### Sample collection

**Fig. 1:**
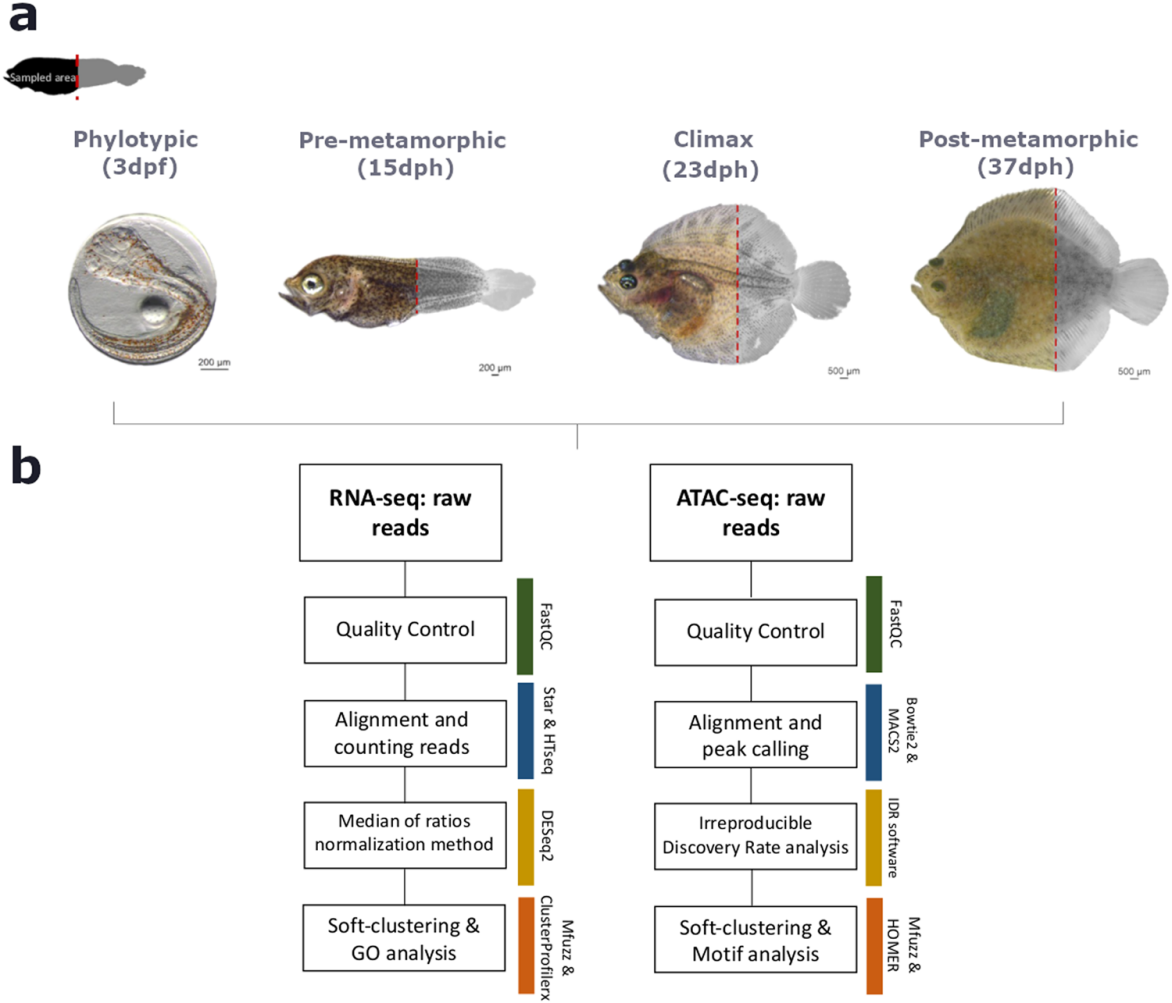
Summary of the experimental method and data analysis. (**a**) Fish samples collected and sampled areas of each turbot stage for RNA-seq and ATAC-seq, from left to right: 3dpf, 15dph, 23dph, 37dph. (**b**) Analysis workflow for RNA-seq and ATAC-seq profiles. dpf, days post fertilization; dph, days post hatching.

Four different stages of turbot metamorphic development were selected: phylotypic (3dpf), pre-metamorphic (15dph), climax of metamorphosis (23dph) and post-metamorphic (37dph) stages, following the morphological criteria described by Al-Maghazachi and Gibson^14^ and Suarez-Bregua^15^ (Fig. 1a). Two independent biological replicates per stage were sampled for RNA-seq and ATAC-seq. Fish were euthanized with a lethal dose of MS-222 (0,25 mg/ml for 30–40 min)^16^ (Sigma-Aldrich, Saint Louis, MO, USA). Specimens from metamorphic stages (15, 23 and 37 dph) were dissected cutting off the last third of the body from the cloaca (Fig. 1a), as a way to eliminate the least variable part of the body. For specimens of phylotypic stage, the whole animal was used. All experimental procedures were approved by the Institutional Animal Care and Use Committee of the IIM-CSIC Institute in accordance with the Spanish regulations (RD53/2013) and European animal directive (2010/63 UE) for the use and protection of experimental animals.

### RNA extraction and sequencing

Up to 30 mg of tissue from each fish (n = 8) was fixed in RNAlater (Thermo Fisher Scientific, USA). Tissue from metamorphic stages (15, 23 and 37 dph) and from whole 3 dpf individuals were disrupted and homogenized in Trizol reagent (Ambion). Total RNA was extracted and purified using the RNeasy Mini Kit (Qiagen) with DNase digestion treatment (Qiagen, Germany). Both RNA concentration and RNA integrity were checked on a Qubit 4 fluorometer (Thermo Fisher Scientific, USA) and Agilent 2100 bioanalyzer (Agilent Technologies, USA), respectively. Libraries were prepared using TruSeq Stranded mRNA LT Sample Prep Kit and sequenced by Illumina HiSeq4000 with paired end 100 bp reads.

### Transcriptome data analysis

Raw reads quality was validated using FastQC v0.11.8 and adapter sequences from the sequencing process were removed using Trimmomatic^17^. Clean reads were mapped to the most current turbot genome assembly (ASM1334776v1) using STAR v2.7.9a software^18^. HTseq-count v2.0.2^19^ was used to calculate the number of aligned reads overlapping each gene and a raw count matrix was generated. The count matrix was used as input data for DESeq. 2 R package v1.26.0^20^ to obtain normalized counts using median ratio method. Hierarchical clustering was perfomed to check the expression pattern along the developmental stages. We selected a set of genes showing specific gene overexpression using Mfuzz R library^21^. In addition, we performed gene ontology enrichment using the R package ClusterProfiler v3.14.3^22^ (Fig. 1b).

### ATAC-seq protocol

Two biological replicates were performed for each stage (n = 8). The 3dpf specimens were rinsed twice with cold 1X PBS and dechorionated with 1 mg/ml pronase in 1X PBS for 4 h at room temperature. Specimens from each developmental stage were rinsed in 1X PBS and tissues were dissociated into individual cells by incubation with 0.125% colagenase in 1X PBS to 37 °C. Incubation times were 9, 20, 40 and 90 min for 3 dpf, 15, 23 and 37 dph turbot. Then, about 75,000 cells cells were lysed by adding lysis buffer (10 mM Tris, pH7.5, 10 mM NaCl, 3 mM MgCl2, 0.1% NP40) and were used for transposition reaction (25 µl, 2x TD buffer, 2.5 µl Transposase and 22.5 µl of nuclease free water) in a total volume of 50 µl for 30 min at 37 °C. Finally, the DNA transposed was purified using a Qiagen MinElute Purification Kit, amplified with primers containing barcodes and purified again with Qiagen MinElute Purification Kit. Libraries were sequenced by Illumina HiSeq4000 with 100 bp paired-end reads.

### ATAC-seq data analysis

A standard pipeline was used to performed ATAC-seq data analysis^11^ (Fig. 1b). Reads were aligned to *S. maximus* genome assembly (ASM1334776v1) using Bowtie2 v2.4.5^23^ with parameters -t–no-unal–no-mixed -X 2000. Position- 4 (reverse strand) or + 5 (forward strand) was determined from the start of the read and extended 5 bp in both directions as the position of the Tn5 cleavage site. MACS2 v2.2.6 software, using the parameters–nomodel–extsize 100–shift −45, was used to perform peak calling and Irreproducible Discovery Rate (IDR) method was used to measure the consistency between replicates. We performed read normalization on the peaks to our ATAC-seq dataset to account for both sample quality and read depth. For clustering ATAC-seq peaks depending on their activity at each stage we used the R package Mfuzz v2.56.0^21^. Motif enrichment analysis of the previously selected peaks clusters was performed using the program FindMotifsGenome.pl form the Homer v.3.1 tool suite.

## Data Records

All datasets from 8 samples belonging to the four developmental stages have been submitted to the NCBI Gene Expression Omnibus (NCBI GEO). Raw files in fastq format and processed files for RNA-seq and ATAC-seq are accessible through the GEO Series accession numbers GSE215395^24^ and GSE215396^25^, respectively.

## Technical Validation

### RNA-sequencing reproducibility control

**Fig. 2:**
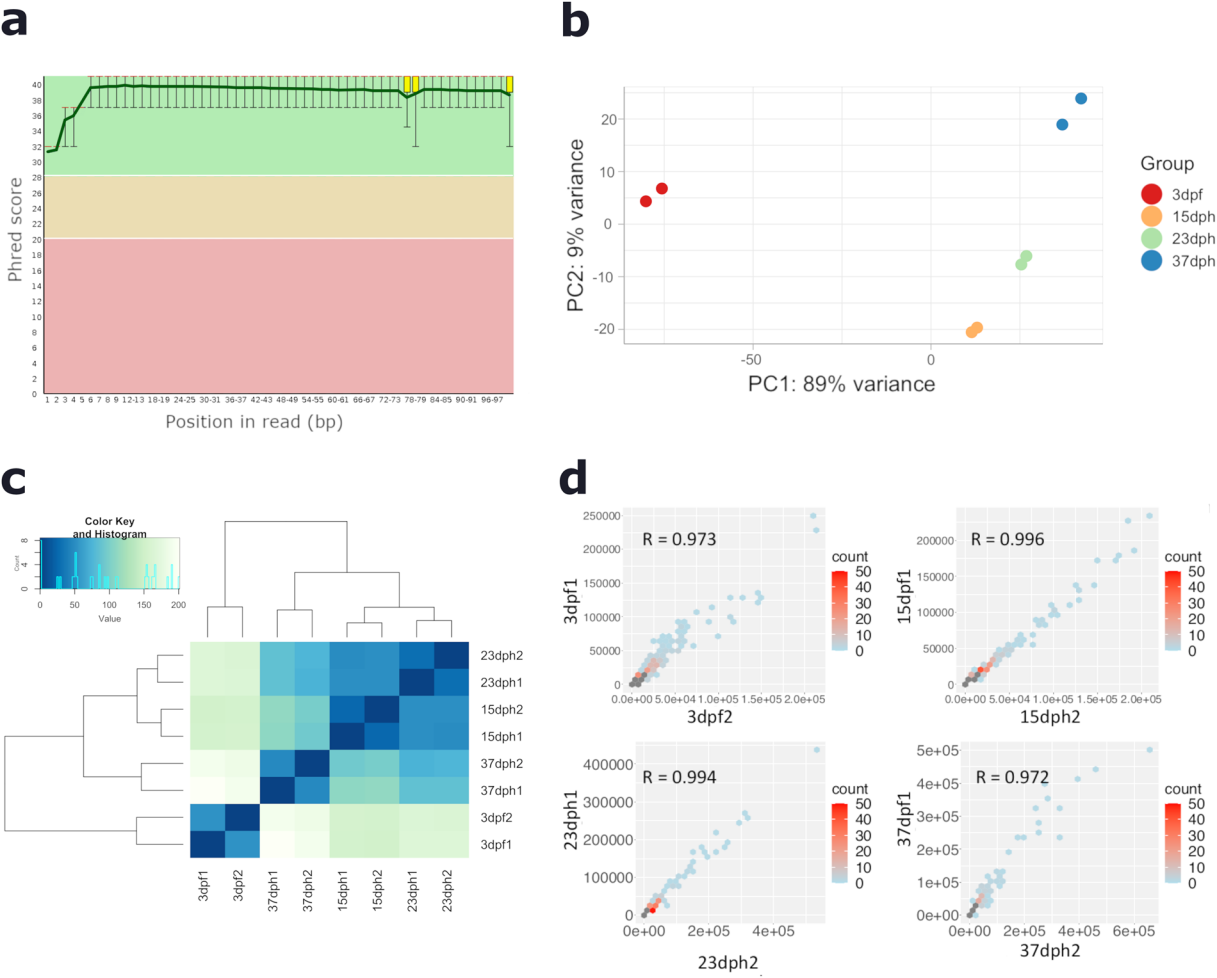
Quality metrics of RNA-seq data. (**a**) Range of quality values across bases at each position of RNA sequencing data. (**b**) PCA plot showing similarities between samples of the 8 RNA-seq profiles. (**c**) Heatmap sample-to-sample distance between the 8 RNA-seq samples. (**d**) Pearson’s correlation between biological replicates.

The number of reads of the sequenced libraries range from 26,101,608 to 33,672,101. Adapters and low-quality sequences were filtered out to ensure good quality analyses, and more than 99% of clean reads were obtained. Reads with a phred quality score above 30 (Fig. 2a), a GC content with a normal distribution and a sequence length of 100 bp were mapped against turbot genome. The mapped sequences ranged from 80% to 90% (Table 1).

**Table 1:** RNA-sequencing summary showing statistical values.

	N raw reads	Clean reads	%Clean reads	N mapped	% mapped
**3dpf1**	29940119	29932601	99.97	27643279	92.35
**3dpf2**	26147847	26143695	99.98	26143695	89.05
**15dph1**	29381469	29379090	99.99	29379090	89.86
**15dph2**	30097489	30095212	99.98	27050421	89.88
**23dph1**	27672397	27670706	99.99	24701121	89.27
**23dph2**	30545076	30542727	99.99	27541761	90.17
**37dph1**	26101608	26093827	99.97	23483848	90.00
**37dph2**	33672101	33668661	99.98	30376727	90.22

After verifying that our samples showed good correlation (Fig. 2b,c and d), we examined the reproducibility of replicates by Pearson’s correlation, obtaining correlation coefficients above 0.9 for all stages (Fig. 2d), revealing a high reliability of the RNA sequencing data. Furthermore, we observed that replicates clustered by stage and, as expected, the earliest stage (3 dpf) was at a greater distance from the rest of the samples (15, 23 and 37 dph) (Fig. 2b,c). The pre-metamorphic (15 dph) and climax (23 dph) stages clustered together indicating that both stages have similar gene expression profiles. This fact suggests that the metamorphosis process at the transcriptomic level started before the morphological changes are detectable.

### ATAC-sequencing quality control

**Fig. 3:**
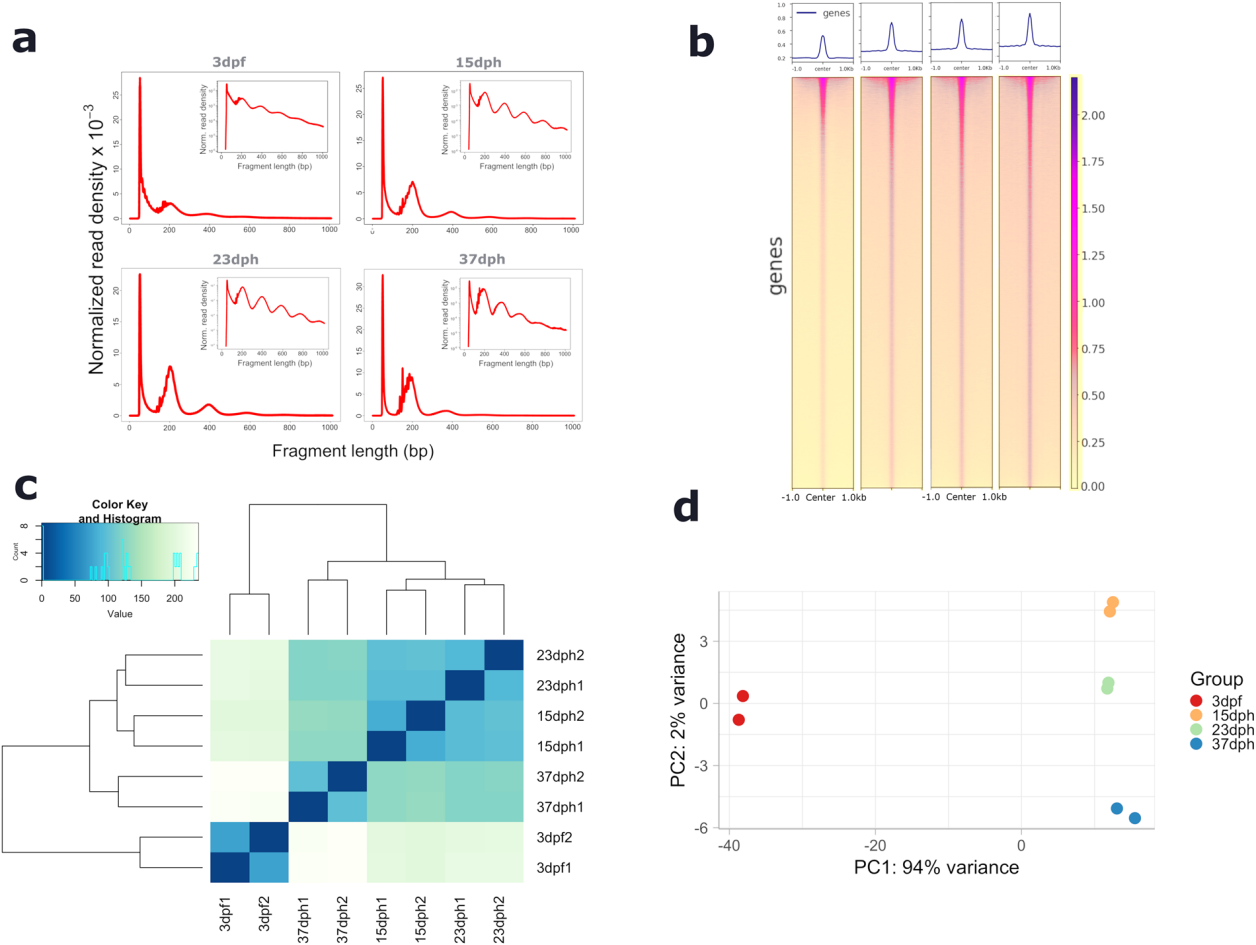
Quality metrics of ATAC sequencing data. (**a**) Fragment size distribution of ATAC sequencing profile at phylotypic, pre-metamorphic, climax and post-metamorphic stages. (**b**) Heatmap of ATAC-seq signal TSS region around 2 kb at 4 stages of turbot developmental. (**c**) Heatmap sample-to-sample distance for ATAC-seq dataset. (**d**) PCA plot showing clustering between 8 ATAC-seq samples based on their similarity.

In Table 2 we present a statistical report of ATAC-seq experiments, including the number of reads, mapping reads and IDR peaks. We evaluated the insert size distribution as an indication of the quality of detection of accessible regions (Fig. 3a). The fragment size distribution exhibits a clear periodicity of approximately 200 bp, corresponding with the position of nucleosomes^26^. Only nucleosome-free reads (fragments < 150 bp) were used for our analyses. Transcription start sites (TSS) were highly enriched in ATAC-seq signal at the four tested stages (Fig. 3b) supporting the good quality of our data. After calling peaks in all replicates using MACS2, we applied the IDR method to identify peaks that are consistent across replicates for each stage. We obtained more reproducible peaks at the earliest stage; the number of peaks is reduced at later stages of development (Table 2). As previously described for RNA-seq samples, replicates correlated well according to their stage, and the earliest stage was also the most divergent. Besides, we also observed a particular similarity between ATAC-seq samples at pre-metamorphic and climax stages (Fig. 3c-d).

**Table 2:** ATAC-sequencing summary showing mapped reads and IDR peaks results.

	N raw reads	Mapped reads	%mapped	IDR peaks
**3dR1**	77642947	66322605	85.42	94714
**3dR3**	73276717	59955010	81.82	94714
**15dR1**	81578826	68852529	84.40	57611
**15dR2**	94320298	77927430	82.62	57611
**23dR1**	70832563	62757651	88.60	47853
**23dR3**	80096654	70236756	87.69	47853
**37dR2**	117213773	99561379	84.94	46248
**37dR3**	99505659	85355954	85.78	46248

### Stage-specific gene expression

**Fig. 4:**
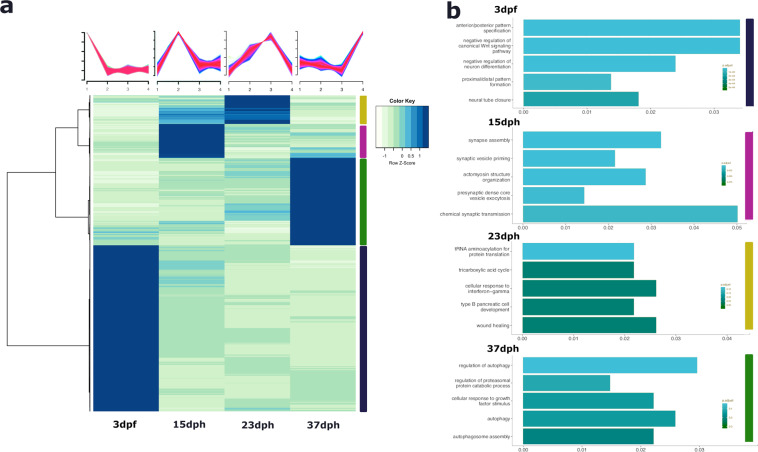
Visualization and analysis of gene expression profile across four developmental stages in turbot. (**a**) Hierarchical heatmap showing the expression pattern of overexpressed genes at each stage. (**b**) Gene ontology enrichment analysis of the clustered genes set depicted in 4a. Color code corresponding to each gene set, blue: 3 dpf; pink: 15 dph; yellow: 23 dph and green: 37 dph.

We could identify stage-specific overexpressed genes using a hierarchical clustering (Fig. 4a). Genes with a peak of expression at phylotypic (1,708 genes), pre-metamorphic (351 genes), climax of metamorphosis (296 genes) and post-metamorphic (898 genes) stages were selected. In an effort to corroborate that our RNA-seq data profile is reproducible, we performed gene ontology analysis with a set of selected genes exclusively up-regulated at each turbot developmental stage (Fig. 4b). The phylotypic stage cluster presents an enrichment of ontology terms such as anterior/posterior pattern specification, in which hox family genes are included, or neural tube closure. Genes up-regulated at pre-metamorphic stage were related to synaptic function (*e.g*., synapse assembly, chemical synaptic transmission) and actomyosin structure organization. Genes up-regulated at the climax of metamorphosis were associated with the immune system (for example with the ontology term of cellular-response to interferon-gamma), in line with other previously published studies^27,28^.

### Stage-specific transcription factor (TF) motifs

**Fig. 5:**
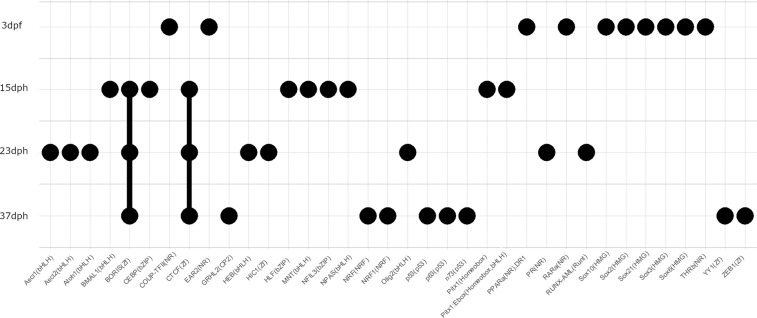
Top 10 transcription factor motifs analyzed at each developmental stage in ATAC-seq profile.

As described above, using a soft clustering tool for our time series data, we selected a unique set of accessible peaks for each developmental stage, obtaining 14,235, 6,516, 3,215 and 5,053 peaks at the phylotypic, pre-metamorphic, climax and post-metamorphic stages, respectively. Subsequently, using Homer software suite, we identified a set of stage-specific TF motifs (Fig. 5). We found that the phylotypic stage does not share any TF motifs with the other stages, but pre-metamorphic, climax and post-metamorphic stages do. Looking more closely, at the phylotypic stage we found TF motifs strongly correlated with early development (*e.g*., SOX family, RARa, THRb). Focusing on pre-metamorphic and climax stages as a single set, we observed interesting overlapping TF motifs: Otx2, involved in eye development as a possible recapitulation of early development to allow eye migration^29^ or Twist2, potential player in the remodeling of existing organs in Xenopus metamorphosis^30^. Motifs for TF important for the activation of myogenesis during metamorphosis (*e.g*., Myf5, MyoG, RUNX) were identinfied in the climax-specific peaks. Finally, following the timeline, chromatin regions accessible at the post-metamorphic stage were associated with p53 family TF motifs. This suggests the need to assure genome stability after a period of change involving new tissue formation and apoptosis processes. Taking together, the reproducibility of replicates, the similarity with RNA-seq results and the stage-specific TF found demonstrate that the atlas of accessible chromatin presented in this work can reliably serve as a reference for future studies.

## Data Availability

We relied on open source tools to perform data analysis. Custom code performed in R used in this analysis have been published in the following repository: https://github.com/GuerreroP/FISHRECAP-ATAC-RNA.
